# Hyperthermic Extracorporeal Applied Tumor Therapy for Six Cycles for Recurrent Metastatic Peritoneal Serous Papillary Carcinoma

**DOI:** 10.7759/cureus.34100

**Published:** 2023-01-23

**Authors:** Roger A Vertrees, Joseph B Zwischenberger, Jan A Winetz

**Affiliations:** 1 Pathology, ®Verthermia Acquisition, Inc., Henderson, USA; 2 Surgery, ®Verthermia Acquisition, Inc., Henderson, USA; 3 Surgery, University of Kentucky, Lexington, USA; 4 Critical Care, ®Verthermia Acquisition, Inc., Henderson, USA

**Keywords:** perfusion hyperthermia, extracorporeal technology, hyperthermia, ovarian cancer, extracorporeal whole-body hyperthermia

## Abstract

An elderly female with failed third-line peritoneal serous papillary carcinoma with metastasis (ovarian cancer) was treated by our proprietary method of whole-body hyperthermia-a recirculating extracorporeal circuit at 42°C for 120 minutes. She received six cycles, 28 days apart. Five index lesions were measured prior to and after each treatment. Results showed stable disease with reduced standard uptake volume. She then restarted six cycles of a previously failed chemotherapy, resulting in no evidence of disease for nine months; she survived for 27 months. Using our technology, the patient experienced an improvement in the quality of life and an increase in survival.

## Introduction

In 2019 there were an estimated 22,240 new cases of ovarian cancer with a mortality of 14,170 cases. This is the fifth most deadly cancer in women and can occur at any age; however, it is most often diagnosed in women older than 50 years of age and is a major cause of death (40-79) [1]. The response rate to first-line therapy is around 80-90%, but most patients relapse and develop chemotherapy resistance with a five-year survival rate of <35% [2]. Survival statistics for recurrent disease are even more dismal with failed third-line chemotherapy having a median survival of 8.9 months (95% CI, 7.8-9.9) [2]. Serous peritoneal papillary carcinoma (SPPC) tumors affect older patients, are more frequently multifocal, exhibit micronodular spread, have a high total load of malignancy in omental and peritoneal surfaces, and are difficult to debulk optimally. Despite effective chemotherapy, SPPC patients survived two to six months less than ovarian cancer patients [3].

Previous studies of perfusion-induced/extracorporeal hyperthermia are limited, mostly due to the difficulty in delivering a precise therapeutic dose and control of electrolytes. Over the past three decades, our group has studied the therapeutic potential of hyperthermia in different research projects: efficacy in cell cultures, mechanisms in molecular biology, rodent models, and safety in swine models that revealed a therapeutic window (TW) for heat centered on 42°C for 120 minutes for cancers derived from solid organs, such as ovarian and lung [4-7]. The result of these investigations is Hyperthermic Extracorporeal Applied Tumor Therapy (HEATT®) which is delivered by our proprietary device, the CoreHFC™ (®Verthermia Acquisition Inc., Henderson, NV, USA), a veno-venous (VV) hyperthermic diafiltration procedure.

Our previous study, an FDA-approved clinical trial, was the first for patients with stage IV non-small cell lung cancer (IDE G960257). In this initial clinical experience, we treated 10 patients with advanced stage IIIB-IV non-small cell lung cancer, using an earlier version of our circuit, veno-venous perfusion-induced systemic hyperthermia (VV-PISH) induced by the Thermochem™ HT-1000 (ThermaSolutions, St. Paul, MN, USA). The hyperthermia was delivered at 42°C average core temperature for 120 minutes. The side effects of treatment were minimal, and results indicated improved patient median survival of 450 days versus concurrent controls of 96 days [8]. The CoreHFC™ circuit is functionally equivalent to the Thermochem™ HT-1000 system with an updated, more efficient dialysis and heat exchanger.

## Case presentation

This patient, a participant in an FDA-approved phase I trial for patients with ovarian cancer, received six repeated doses of HEATT®, 28 days apart. Her diagnosis was stage IV peritoneal serous papillary carcinoma with metastasis. She was a 67-year-old Latina female who was admitted directly from hospice care, was morbidly obese, but was able to perform normal activities with effort. She had five lesions that qualified as index lesions (RECIST 1.1 criteria) [9] and were identified and measured at baseline, prior to her first cycle, and 21 days after each treatment. These were a palpable pelvic tumor mass, a hypermetabolic soft tissue mass in the right psoas and iliacus muscles, a hypermetabolic soft tissue mass along the left common and external iliac arteries, a left para-aortic lymph node, and a mediastinal lymph node. She was treated previously for uterine cancer; other past cancer-related therapies included laparotomy with tumor debulking, gemcitabine (twice), carboplatin, Avastin and Taxol, and external beam radiation. Her comorbidities were swelling of her feet and ankles, musculoskeletal weakness, diabetes, a deep vein thrombosis requiring an iliac venous stent in her right leg, and incapacitating leg pain from nerve root compression of L4 and S1. She had failed third-line chemotherapies and had a predicted median survival of 8.9 months [2]. Patients under hospice care are expected to survive less than six months [10].

Results of HEATT®

Table 1 displays the results of the intraoperative heating. The cycles were 28 days apart for six months. Before HEATT® her Karnofsky score was 80. During HEATT® there were both an improvement in her Karnofsky score from 80 to 100 and in her overall well-being. Therefore, she was able to resume vigorous daily activities, as well as travel outside of the United States to visit family.

**Table 1:** Operative Outcomes *Thermal Therapeutic Window (TTW) **Length of stay (LOS) in hospital after the day of the procedure ***Karnofsky score as assessed 28 days after each cycle of HEATT®. (Note a) she performed a deep cleaning of her home, first time since cancer diagnosis (Note b) she traveled outside the US to visit family. ****Adverse Events at the time of discharge: (1) nausea, vomiting, and diarrhea (2) thrombocytopenia (3) elevated SGOT/SGPT

Cycle #	TTW*	LOS**	Karnofsky Score***	Adverse Events at Discharge****
1	Y	3	90	1
2	Y	2	100	2,3
3	Y	2	100 (Note a)	2
4	Y	1	100 (Note b)	2
5	Y	2	100	1,2,3
6	Y	3	100	1,2

Post-HEATT® results

**Figure 1:**
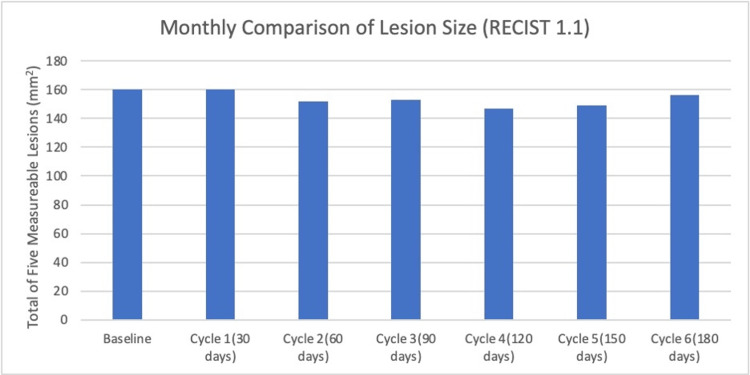
Lesion Size Comparison

Figure 1 shows values obtained from Computed Tomography (CT) scans that were performed 21 days after HEATT®, analyzed by RECIST 1.1 criteria [9], and then summed over the five index lesions. These data indicate no significant change in the overall size of the five measured lesions over the six months of HEATT®, implying stable disease.

**Figure 2:**
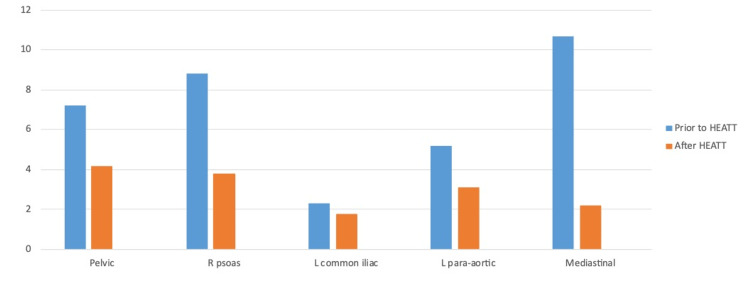
Standard Uptake Value (SUV)

Figure 2 demonstrates standard uptake value (SUV) comparisons before and after the six HEATT® treatments for all five lesions as demonstrated by Positron Emission Tomography-Computed Tomography (PET-CT). The SUV measured the metabolic activity in the index lesions, and the average results are compared between the patient’s baseline and the final value after all six HEATT® exposures for all five lesions. The results demonstrate a 65% overall reduction in the SUV.

**Figure 3:**
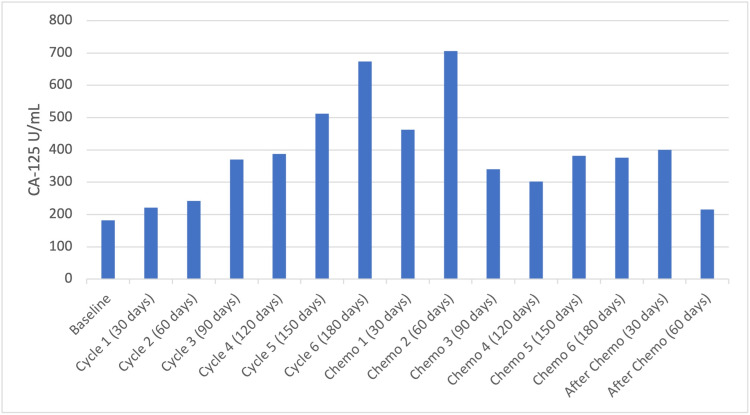
CA-125 (NR <35 U/mL) Time Course

The patient’s CA-125 values were measured after each cycle of HEATT® and after follow-up chemotherapy treatments with the overall results shown in Figure 3. Her CA-125 values continued to increase during the HEATT® treatments and then fell drastically (percent change is 88%) during her follow-up chemotherapies. CA-125 is a component of the ovarian cancer cell membrane, epitope within the MUC 16 [11]. Since the CA-125 values increased because of the HEATT® treatment, this may be interpreted as a sign of destruction of the cancer cell membrane.

Posttreatment follow-up

**Figure 4:**
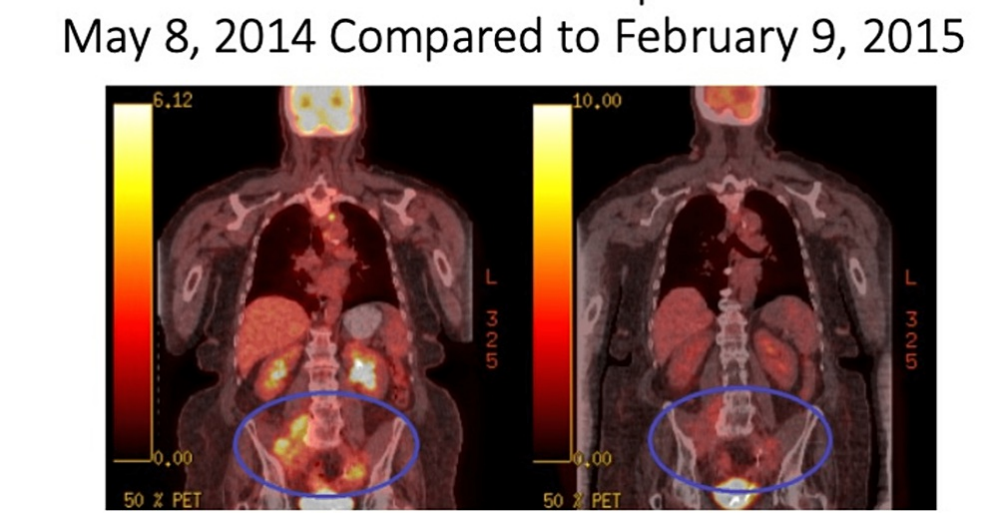
PET-CT Comparisons Before and Six Months After Completion of HEATT® and Chemotherapy Regimens Comparison between PET-CT prior to HEATT® (left side) and after both HEATT® and chemotherapy (right side).

After the patient’s final HEATT®, she had symptomatic improvement of her leg pain and no longer used a cane. There was no palpable tumor on pelvic exam, she had no edema and a normal neurological exam of her left extremity, and her weight was stable. Imaging by PET-CT showed a 65% reduction in tumor metabolism (SUV). After completing six cycles of HEATT®, she restarted chemotherapy the following month, utilizing Avastin, carboplatin, and gemcitabine for six months. This treatment regime provided approximately nine months of no evidence of disease (NED) as shown in Figure 4. This was the same chemotherapy regimen that she had failed five months prior to entry into the trial.

Nineteen months after HEATT®, tumor growth was detected with a new tumor on the left side of her neck. The patient had increased pain in her right knee and decreased sensation in her hands and fingers at night, which was associated with the chemotherapy. Her Karnofsky score was 80. Twenty-two months after HEATT®, there was a significant advancement of her tumor. She had increased right knee pain and dysesthesia, a decrease in appetite, and lethargy. She was put in hospice care; her Karnofsky score was 50 at this time. The patient died from urosepsis 27 months after progression of disease after having six rounds of HEATT® and a return to previously failed chemotherapies. The risk-benefit, the overall median expected survival (E) and observed survival (O), for this patient was 8.9 months and 27 months, respectively, for an O/E of 27/8.9=3.03. This strongly supports the use of HEATT® in patients with metastatic ovarian cancers [10].

## Discussion

This patient was an elderly female with stage IV peritoneal serous papillary carcinoma with metastasis who was admitted directly from hospice. During HEATT® treatments, she received no other types of antitumor therapy. Using RECIST 1.1 criteria [9], she maintained stable disease and showed a decrease in the metabolic activity (SUV) of her index tumors. After completion of the six-month HEATT® trial period, she returned to six months of the previous chemotherapy regimen of Avastin, carboplatin, and gemcitabine. Following the six months of chemotherapy, she displayed no evidence of disease for nearly nine months as shown in the PET-CT. Twenty-two months after the conclusion of HEATT®, there was a significant advancement of her tumor. She died from urosepsis 27 months after progression of disease.

Induced hyperthermia, ≥42°C for 120 minutes, causes a physical alteration in the body’s homeostasis and may manipulate the tumor microenvironment (TME) to be more readily available to subsequent therapies. The TME results from the unique characteristics of the tumor vasculature. Tumor vasculature is more permeable because of a lack of smooth muscle cells leading to a ‘leaky’ endothelial cell lining and is chaotic in distribution. This vascular disarray leads to regions that are hypoxic resulting in anaerobic glycolysis, inadequate removal of waste products, and acidosis [12-13]. More precisely, the TME consists of cellular components, the extracellular matrix (ECM) and soluble products that protect the tumor and thwart antitumor strategies. There is an increasing awareness of the usefulness of hyperthermia when part of an integrative approach to treating patients with cancers [14]. Hyperthermia has been shown to increase tumor blood flow and the perfused fraction of the tumor [15]. Accompanying this increased blood flow are enhanced vascular permeability, increased oxygenation, decreased interstitial fluid pressure, and normalizing of the pH. Additionally, hyperthermia has been shown to increase immune cell trafficking into tumors [16], regulate lymphocyte trafficking [17], alter cytokine activity [18], increase metabolic rate [19], and upregulate gene expression favoring apoptosis [5].

## Conclusions

Prior to this report, none of the desirable effects attributed to systemic hyperthermia have been studied specifically in peritoneal serous papillary carcinoma with metastasis. The patient received six repeated doses of HEATT®, met the safety criteria after each cycle, and was able to qualify for the next round. Following six cycles of HEATT®, she resumed chemotherapy, sustained stable disease, and achieved increased activity and survival beyond what was predicted. We recommend continued clinical application of HEATT® in advanced metastatic cancers that are unresponsive to chemotherapy and/or immunotherapy as part of an integrative approach to comprehensive care.
